# How Accurate Is MOLLI T1 Mapping In Vivo? Validation by Spin Echo Methods

**DOI:** 10.1371/journal.pone.0107327

**Published:** 2014-09-11

**Authors:** Mitchell A. Cooper, Thanh D. Nguyen, Pascal Spincemaille, Martin R. Prince, Jonathan W. Weinsaft, Yi Wang

**Affiliations:** 1 Department of Biomedical Engineering, Cornell University, Ithaca, New York, United States of America; 2 Department of Radiology, Weill Cornell Medical College, New York, New York, United States of America; 3 Division of Cardiology, Department of Medicine, Weill Cornell Medical College, New York, New York, United States of America; University of California San Francisco, United States of America

## Abstract

T_1_ mapping is a promising quantitative tool for assessing diffuse cardiomyopathies. The purpose of this study is to quantify in vivo accuracy of the Modified Look-Locker Inversion Recovery (MOLLI) cardiac T_1_ mapping sequence against the spin echo gold standard, which has not been done previously. T_1_ accuracy of MOLLI was determined by comparing with the gold standard inversion recovery spin echo sequence in the calf muscle, and with a rapid inversion recovery fast spin echo sequence in the heart. T_1_ values were obtained with both conventional MOLLI fitting and MOLLI fitting with inversion efficiency correction. In the calf (n = 6), conventional MOLLI fitting produced inconsistent T_1_ values with error ranging from 8.0% at 90° to 17.3% at 30°. Modified MOLLI fitting with inversion efficiency correction improved error to under 7.4% at all flip angles. In the heart (n = 5), modified MOLLI fitting with inversion correction reduced T_1_ error to 5.5% from 14.0% by conventional MOLLI fitting. This study shows that conventional MOLLI fitting can lead to significant in vivo T_1_ errors when not accounting for the lower adiabatic inversion efficiency often experienced in vivo.

## Introduction

Myocardial T_1_ mapping is a non-invasive MRI based tissue characterization technique that uses measurements of the T_1_ relaxation time in the heart for diagnosis and treatment. T_1_ mapping has been applied to a broad range of cardiac applications including quantification of lipid or iron deposition in the myocardium, and detection of subtle pathological changes due to edema [1]. In addition, T_1_ mapping shows a great promise for assessing cardiac amyloidosis [2]–[4] and other non-ischemic or congenital cardiomyopathies that result in diffuse myocardial fibrosis [5]–[9]. The MOLLI (Modified Look Locker Inversion Recovery) sequence [10] is a fast 2D inversion recovery (IR) balanced steady-state free precession (bSSFP) based T_1_ mapping method for cardiac imaging that is increasingly being used to probe differences between healthy and diseased states in the myocardium. While the accuracy of MOLLI has been studied extensively using computer simulations and water phantoms [10]–[13], a direct comparison of MOLLI with the gold standard IR spin echo (IR-SE) method has not yet been performed in vivo, where tissues may behave differently from water phantoms. This work aims to quantify MOLLI accuracy in vivo by comparing with the accurate but time-consuming gold standard IR-SE sequence in the calf muscle and with a rapid IR fast spin echo (IR-FSE) sequence in the heart of healthy volunteers. In addition, we quantified the effect of correcting for imperfect inversion efficiency [13]–[15] on the T_1_ accuracy of MOLLI.

## Materials and Methods

### T_1_ Fitting Approaches for MOLLI Data

**Figure 1:**
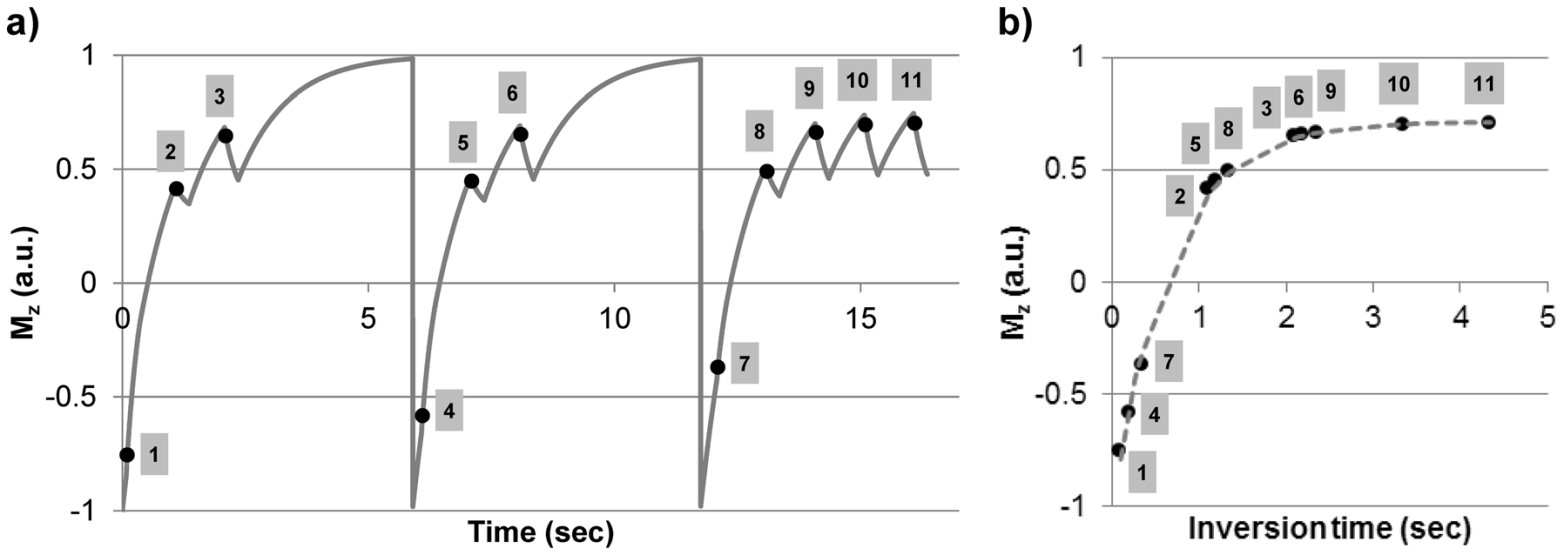
MOLLI acquisition and data fitting: a) Simulated longitudinal magnetization during MOLLI acquisition (T_1_ = 1000 ms, T_2_ = 30 ms, readout flip angle = 30°, echo train length = 64, heart rate = 60 bpm). Note the complex pattern of the underlying magnetization evolution due to mixed periods of bSSFP readout and free relaxation. MOLLI data are sampled at 11 inversion times marked; b) Conventional MOLLI fitting approximates the rearranged MOLLI data with a mono-exponential function to derive an apparent T_1_, which is then corrected according to Eq.1.

The MOLLI sequence consists of inversion pulses followed by mixed periods of bSSFP readout and free relaxation and therefore has a fairly complex signal evolution (Fig.1). To overcome this problem, previous T_1_ mapping studies using MOLLI typically approximated this signal curve using a mono-exponential fit to extract an apparent T1 relaxation time (T_1_^*^) and subsequently corrected it to yield an improved T_1_ estimate [10]:
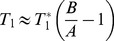
(1)where T_1_^*^ (the apparent T_1_ relaxation), A and B are obtained by a three-parameter exponential fit of the MOLLI data (after restoring signal polarity and rearranging data according to their inversion times). While simple, this correction was originally derived for T_1_ mapping with IR spoiled gradient echo (SPGR) Look-Locker imaging with continuous readouts [13], [16] and therefore is not directly applicable to IR-bSSFP *modified* Look-Locker imaging with mixed readout and free relaxation periods. As a result, the correction in Eq.1 is known to deliver accurate T_1_ estimates only for certain T_1_/T_2_ values and flip angles [11]–[13].

In addition to not fully accounting for complex MOLLI signal evolution (Fig.1), Eq.1 assumes perfect inversion efficiency (i.e., 100% of the longitudinal magnetization is inverted by the inversion pulse) [13]–[15]. Typically, long adiabatic inversion pulses (e.g., the hyperbolic secant pulse is approximately 8 ms on our system) are favored over short hard inversion pulses to provide uniform inversion in the presence of B_0_ and B_1_ field inhomogeneities in vivo [10], [17]. These adiabatic pulses can introduce non-negligible T_2_ induced signal loss in tissues with shorter T_2_ relaxation times such as muscle and myocardium. It is possible to account for this error when the inversion efficiency is known. Reference [15] derived the following equation from Eq.1 to include inversion efficiency:
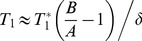
(2)where δ is the measured inversion efficiency. However, to our knowledge this has only been tested in phantoms [15] and has yet to be demonstrated in vivo. In this work, the inversion efficiency was obtained as a by-product of the T_1_ fitting of the gold-standard IR-SE data and used in the fitting of MOLLI data.

### Imaging Experiments

All experiments were performed on a 1.5T GE HDxt MRI scanner (GE Healthcare, Waukesha, WI) using an 8-channel receiver coil (GE Healthcare Coils, Aurora, OH). The study was approved by the Weill Cornell Institutional Review Board and all healthy volunteers provided written informed consent prior to imaging. Due to the length of the MRI study in the calf, healthy volunteers were enrolled in two separate groups for the calf and cardiac imaging experiments (one volunteer participated in both experiments). A 2D IR-SSFP MOLLI sequence was implemented following the method described in [10]. An 8.12 ms hyperbolic secant adiabatic inversion pulse and a 0.5 ms apodized half-sinc SSFP excitation pulse were used for all experiments.

MOLLI data was acquired in the calf muscle of 6 volunteers (5 men, 1 woman, mean age 30±6 years). Calf muscle was chosen for in vivo validation since its MR relaxation properties (T_1_/T_2_ ∼1000/30 ms) are similar to that of myocardium (T_1_/T_2_ ∼1100/50 ms). Imaging the calf muscle also eliminates the confounding effects of motion and heart rate variability on the T_1_ accuracy of MOLLI [18], [19], as well as enables the acquisition of the time-consuming gold standard IR-SE data, which is impractical in the heart. Typical MOLLI imaging parameters were as follows: TR = 4.1 ms, TE = 1.2 ms (asymmetric echo), matrix = 256×128 (interpolated to 256×256), FOV = 26–30 cm, partial FOV factor = 0.5, 11 inversion times (TI) = 100, 200, 350, 100+RR, 200+RR, 350+RR, 100+2RR, 200+2RR, 350+2RR, 350+3RR, 350+4RR (RR  =  cardiac interval). A 3 sec free relaxation period was introduced between subsequent modified Look-Locker experiments similar to that used in the original MOLLI sequence [10]. A 6 Kaiser-Bessel RF ramp [20] was used to prepare magnetization prior to SSFP data acquisition. MOLLI data were acquired with 30°, 60° and 90° readout flip angles. 2D IR-SE reference data was acquired with the following imaging parameters: TR = 6 sec, TE = 10 ms, matrix = 256×128 (interpolated to 256×256), partial FOV factor = 0.5, TI = 20, 300, 1000 ms and ∞, scan time = 26 min. Synthetic ECG gating signal (80 bpm) provided by the scanner software was used to avoid variations in the cardiac R-R interval.

Cardiac MOLLI data were acquired in 5 healthy volunteers (5 men, mean age 36±11 years) with peripheral gating and imaging parameters similar to that reported in [11], [21]: TR = 3.3 ms, TE = 0.9 ms (asymmetric echo), flip angle = 30°, FOV = 35–36 cm, partial FOV factor = 0.75, parallel imaging (ASSET) factor R = 2, 11 TIs, 3 heartbeat pause between subsequent modified Look-Locker experiments, scan time = 17 heartbeats. Since the gold standard IR-SE sequence is too time-consuming for cardiac applications, a rapid cardiac gated single-shot IR-FSE sequence with variable refocusing flip angles was implemented to obtain reference myocardial T_1_ within one breath-hold [22]. The typical IR-FSE imaging parameters were as follows: TR = 5 RR, minimum TE = 4 ms, echo train length = 48, matrix = 256×128, partial FOV factor = 0.75, TI = 20, 300, 1000 ms and ∞, ASSET factor R = 2, scan time = 16 heartbeats. Before use as a reference in the heart, the accuracy of the IR-FSE sequence was verified against the gold standard IR-SE in the calf muscle.

### Data Analysis

All data were processed using Matlab R2009a (The Mathworks, Natick, MA) on a Dell XPS 8100 desktop computer. The polarity of the sampled data was restored using the method proposed in [23]. IR-SE data were fit using a three-parameter exponential signal equation A – B x exp(-TI/T_1_) to obtain the reference T_1_ and inversion efficiency (δ  =  B/A – 1). MOLLI data were fit using both conventional MOLLI fitting (Eq.1) and MOLLI fitting corrected with known inversion efficiency (Eq.2). Equation 2 used the inversion efficiency δ obtained from IR-SE acquisition.

In the calf, signals were averaged within a 3×3 region of interest (ROI) placed in the right soleus muscle of each volunteer prior to data fitting. There were no repeated measurements in the left and right calf muscles of the volunteers. For cardiac imaging, pixel-wise fitting was performed for an ROI placed in the left ventricular septum wall. IR-FSE data were processed using a three-parameter exponential fit to obtain the reference T_1_.

Ideally one needs to perform mapping of inversion efficiency in the heart in order to accurately correct for myocardial T_1_ obtained by MOLLI. Because this by itself is a difficult problem (e.g., due to respiratory and cardiac motion), a partial solution was pursued by mapping the inversion efficiency in the calf muscle and then adjusting this value for the heart muscle based on the theoretical difference predicted by Bloch simulation while accounting for expected values of T_1_ and T_2_ in the myocardium (see Results section).This allowed us to demonstrate the improved T_1_ accuracy that can be obtained in the heart with inversion efficiency correction.

Relative T_1_ error defined as |T_1,MOLLI_-T_1,IRSE_|/T_1,IRSE_ × 100 and relative fitting residual defined as ||S_measured_-S_fitted_||_2_/||S_measured_||_2_ × 100 were calculated, and statistical significance of the difference between the mean measured T_1_ and the mean T_1_ from IR-SE/FSE (gold standard) was determined using a one-way analysis of variance (ANOVA) followed by Tukey's post-hoc test for multiple comparison. A P-value of less than 0.05 was considered statistically significant.

## Results

**Figure 2:**
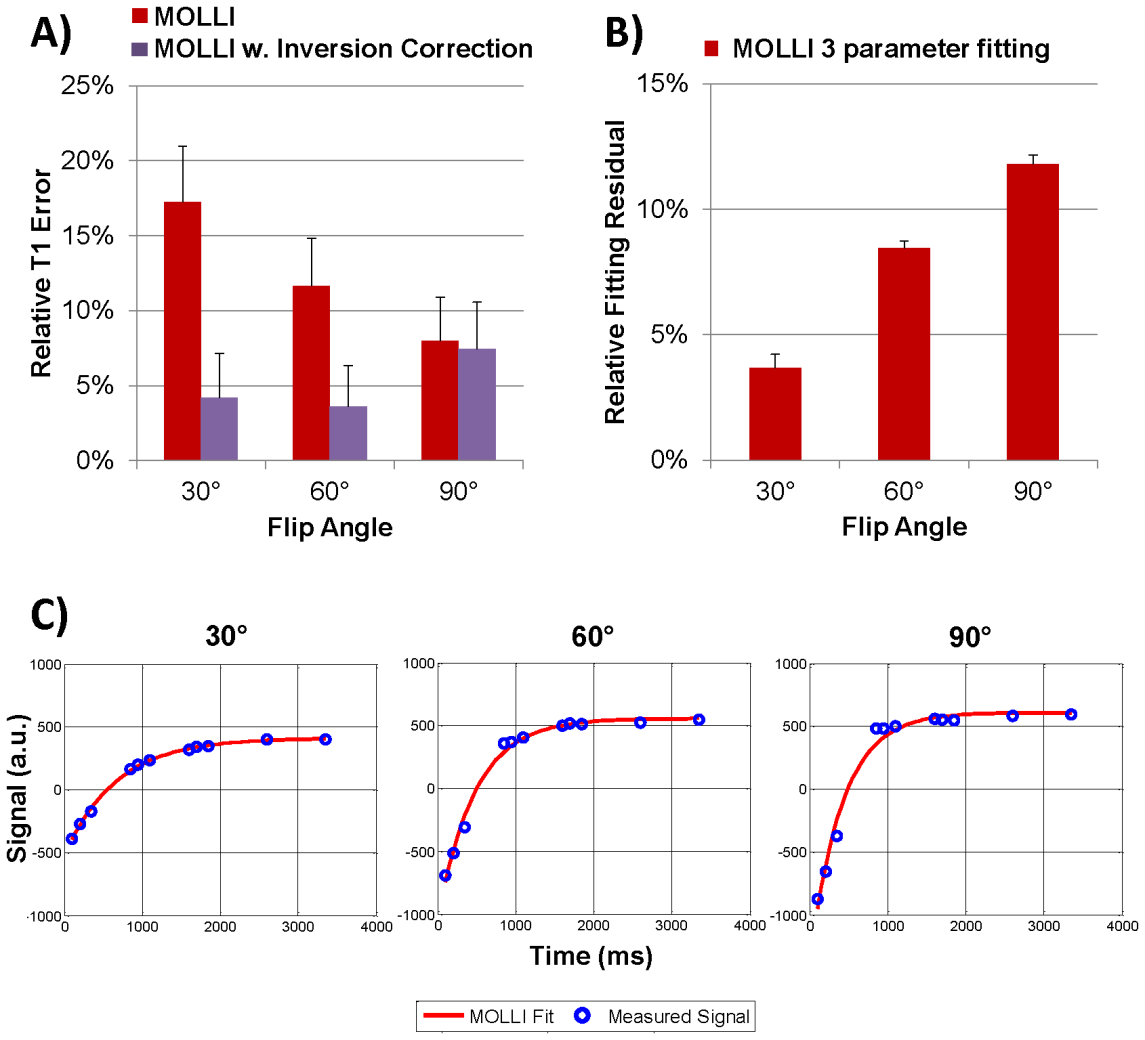
MOLLI results in the calf muscle (n = 6): A) T_1_ errors obtained at 30°, 60° and 90° readout flip angle for conventional MOLLI fitting (Eq.1) and MOLLI fitting with inversion correction (Eq.2); B) Relative fitting residuals for the three parameter fit used in Eqs.1 and 2. Note the increasing residuals with the three parameter fitting (used by Eqs.1 and 2) at higher flip angles; C) Measured signal and curves fit with the 3-parameter MOLLI fit in the calf muscle of one volunteer at 30°, 60° and 90°. Notice the increasing discrepancy between the fit and measured data at higher flip angle, confirmed by the increase in fitting residual in (B).

In the calf muscle (n = 6), conventional MOLLI fitting (Eq.1) produced fairly inaccurate and inconsistent T_1_ values as the readout flip angle was varied, with average T_1_ error reaching as high as 17.3% at 30° (Fig. 2a/Table 1). Interestingly, T_1_ error by conventional MOLLI fitting was lower at higher flip angles (reducing to 8.0% at 90°) although the relative fitting residual increased markedly (Fig. 2b), from 3.7% at 30° (indicating a reasonable fit of the 3-parameter exponential model) to 11.8% at 90° (indicating a rather poor fit as shown in Fig. 2b/c). Using MOLLI fitting accounting for the inversion efficiency (Eq.2), error was improved to be less than 7.4% at all flip angles.

**Table 1:** Comparison of T1 values obtained in the calf muscle (n = 6) and myocardium (n = 5) of healthy volunteers using conventional MOLLI fitting (Eq.1) and MOLLI fitting with inversion correction (Eq.2) at various flip angles (FA).

Calf Muscle (n = 6)								
			FA = 30°		FA = 60°		FA = 90°	
	IR-SE	IR-FSE	MOLLI	MOLLI w. Inv	MOLLI	MOLLI w. Inv	MOLLI	MOLLI w. INV
T1 (ms)	986±32	987±42	815±40	952±38	871±36	1016±27	907±35	1058±27
Inv. Eff.	85.7±1.6%	81.9±2.0%	N/A					
P	N/A	1	<0.001	0.69	<0.001	0.79	0.007	0.02
Fitting residual			3.7±0.6%		8.5±0.3%		11.8±0.36%	

Myocardium (n = 5)			
		FA = 30°	
	IR-FSE	MOLLI	MOLLI w. Inv
T1 (ms)	1092±64	937±60	1065±68
Inv. Eff.	88% (sim. from calf and Bloch)	N/A	
P	N/A	0.006	0.78
Fitting residual		5.3%±1.8%	

P values are given for comparison with the gold standard IR-SE method (calf) or IR-FSE method (myocardium).

**Figure 3:**
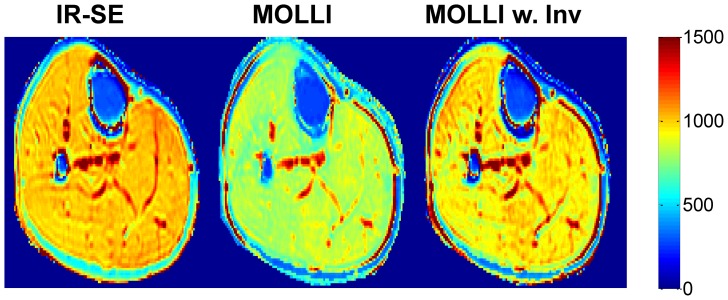
Example of T_1_ maps (in ms) obtained with IR-SE, conventional MOLLI fitting and MOLLI fitting with inversion correction in the calf muscle at 30° flip angle.

Figure 3 shows an example of T_1_ maps obtained in the calf muscle at 30°. The average inversion efficiency g obtained from IR-SE and used in MOLLI data fitting was 85.7±1.6%. The average fitting time per pixel was 0.03 s for three-parameter MOLLI fitting, assuming that the polarity of the signal curve had already been restored.

In the calf muscle, the gold standard IR-SE and rapid IR-FSE acquisitions were found to provided similar T_1_ values (1.3±0.7% relative error). However, the inversion efficiency measured by IR-FSE (81.9±2.0%) underestimated that obtained by IR-SE (85.7±1.6%) p = 0.002. To determine the in vivo inversion efficiency in the myocardium required for MOLLI data fitting, the theoretical inversion efficiency was simulated using T_1_/T_2_ of healthy soleus muscle (985/31 ms) [24] and myocardium (1088/52 ms) [22], [25] and the shape of the adiabatic pulse used in the imaging experiment. Since simulation may not perfectly predict the in vivo inversion efficiency [15], the ratio of the simulated inversion efficiencies was then used to scale the average inversion efficiency obtained from the calf muscle (85.7%), yielding an inversion efficiency estimate of 88% for fitting of the cardiac data. B_1_ and B_0_ effects were not considered when estimating the inversion efficiency since the hyperbolic secant adiabatic inversion pulse is expected to be robust against B_1_ and B_0_ inhomogeneity encountered in vivo at 1.5 T. For T_1_ and T_2_ similar to that of the calf and the myocardium, the inversion efficiency is expected to vary by only a few percent with ± 25% variation in RF amplitude and ± 150 Hz off-resonance range as shown in Fig.4 of [15].

**Figure 4:**
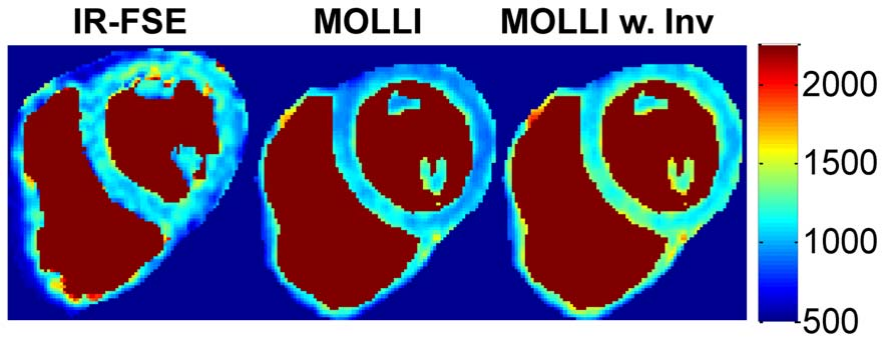
Example of myocardial T_1_ maps (in ms) obtained with IR-FSE, conventional MOLLI fitting and MOLLI fitting with inversion correction at a 30° flip angle. The IR-FSE image was taken in a separate breath-hold than the MOLLI and therefore it is at a slightly different position. Blood has been segmented out to minimize distraction due to the blood T_1_: IR-FSE spoils the blood signal and is not able to fit for blood T_1_, however MOLLI is able to fit for T_1_ in the blood.

In the heart (n = 5), MOLLI with inversion correction (T_1_  =  1065 ± 68 ms; p = 0.78) reduced T_1_ error compared to conventional MOLLI fitting (T_1_  =  937 ± 60 ms; p = 0.006) when compared to the reference T_1_ values provided by IR-FSE (T_1_  =  1092 ± 64 ms). Overall, the relative T_1_ error was 14.0±6.6% for conventional MOLLI fitting, and 5.5±5.1% for MOLLI with inversion efficiency correction, closely following the trend observed in the calf experiments. A comparison of short-axis myocardial T_1_ maps obtained with the two MOLLI data fitting methods is shown in Fig.4. The average heart rate was 52.8±9.6 bpm (range 40–63 bpm).

## Discussion

Our in vivo data showed that conventional MOLLI (Eq.1) provided fairly inaccurate and inconsistent in vivo T_1_ values in muscle tissues when compared with the standard spin echo based methods. As indicated by our results, a major source of T_1_ error comes from reduced adiabatic inversion efficiency due to shorter T_2_ of muscle tissues (T1/T2 ratio ∼30). The correction for apparent T_1_ used in the conventional MOLLI fitting was originally derived for SPGR Look-Locker imaging assuming 100% inversion efficiency and therefore may not be adequate for describing a more complicated bSSFP signal evolution in MOLLI (Fig.1), especially at reduced inversion efficiency. Limitations of the model are shown in figure 2 when imaging at higher flip angles causes larger fitting residuals. When accounting for the inversion efficiency in conventional MOLLI fitting (Eq. 2), T_1_ error was greatly reduced. The current work shows that conventional fitting after inversion efficiency correction (Eq. 2) is valid, even though, strictly speaking, it only applies to spoiled gradient echo based Look-Locker acquisitions [13], [16]. This finding agrees well with recent results obtained with an SPGR based MOLLI sequence at 7T [14] and serves to confirm that a source of error with traditional MOLLI T1 mapping is the inversion efficiency, which can be improved upon when using Eq. 2 if the inversion efficiency is measured. Further in vivo studies may be warranted to quantify T_1_ error in MOLLI as a function of inversion efficiency in different tissues.

The conventional MOLLI fitting method (Eq.1) has been validated in water phantoms doped with various contrast agents [10]–[12], [18] by comparing with the gold standard IR-SE method. However, water phantoms are different from in vivo tissue in biochemical composition and underlying biophysical process (e.g., exchange among water compartments), resulting in different relaxation and MRI signal behavior. In vivo validation of MOLLI has not been performed so far most likely due to the excessive IR-SE acquisition time. In this work, we used the calf muscle as a tissue model for the myocardium, thus enabling for the first time a direct in vivo comparison of MOLLI with IR-SE. For cardiac T_1_ mapping, a more rapid IR-FSE sequence was developed to enable cardiac T_1_ mapping in a single breath-hold [22]. While conventional MOLLI fitting of phantom data traditionally shows good to excellent T_1_ accuracy [10]–[12], [18], we found larger in vivo T_1_ errors (above 10%) in both the calf muscle and the myocardium which could be reduced to less than 7.4% when accounting for inversion efficiency as in Eq.2. This observation suggests that in vivo validation should be considered following the initial phantom validation when assessing the performance of T_1_ mapping for in vivo imaging.

Our in vivo T_1_ results obtained with the conventional MOLLI fitting (Eq.1) are similar to that reported in previous studies (761 ms in the skeletal muscle [10] and 962–998 ms in the myocardium [10], [18], [21]). Interestingly, we observed increasing T_1_ values at higher readout flip angles in the calf muscle, a trend opposite to that reported previously in the myocardium [11]. To better understand this phenomenon, we have performed Bloch simulation for T_1_/T_2_ of the calf muscle and the heart and observed that the trend highly depends on tissue T_2_ and other timing parameters such as heart rate. In addition, conventional MOLLI analysis (Eq.1) does not explicitly take into account the flip angle, since it was originally derived for SPGR imaging, and as a result can provide a flip angle dependent bias specific to tissue T1/T2. This bias may explain the increase in T1 error after inversion efficiency correction, especially at higher flip angles of 60° and 90°. This is additional evidence that the conventional MOLLI data fitting may not work well for different tissues or when imaging conditions are changed.

The above findings are clinically relevant for two reasons. First, inversion efficiency correction can significantly reduce T_1_ error in MOLLI to within 7.4%, thereby improving the reliability of this technique for diagnostic purposes. Second, the improved consistency of T_1_ estimates at different flip angles and T_1_/T_2_ values after applying correction (Eq. 2) to MOLLI analysis may benefit multi-site multi-vendor T_1_ mapping studies by reducing discrepancies due to different hardware and software implementations.

This work has several limitations. The effect of inversion efficiency on T_1_ fitting post contrast, which is important for clinical application, was not investigated. This was because myocardial T_1_ can vary significantly post contrast depending on factors such as cardiac output and bolus timing, making accurate comparisons between the reference IR-FSE sequence and the MOLLI sequence in multiple subjects challenging. The long echo train of the IR-FSE sequence may also result in errors due to blurring in tissue with short T_1_. The trade-off between scan time reduction by prolonging the echo train (which is relevant for breath-hold cardiac imaging) and the associated image blurring in such situations is an important question and will be investigated in our future work. In addition, post-contrast myocardial T_2_ is difficult to measure for the same reason, making the estimation of inversion efficiency non-trivial. In this work, in vivo inversion efficiency was obtained as a by-product of IR-SE data fitting, which is not a practical method due to long IR-SE acquisition time and the lack of experimental mapping of in vivo inversion efficiency in the heart further limited the work.

A fitting method that can improve MOLLI T1 estimates in vivo using Eq.2 would require the knowledge of imaging and tissue parameters (e.g., inversion efficiency), which may not be readily available. Mapping inversion efficiency, for example, is a non-trivial problem particularly in the in vivo setting, and this is an important limitation of the MOLLI approach. Another possible avenue for inversion efficiency correction in MOLLI would be to measure the inversion efficiency in the calf in an initial study in both healthy subjects and patients, and then establish and validate a population average. We believe however that IR-FSE is a better approach in patients with lower heart rate (to reduce the motion sensitivity of the FSE readout) and when blood T_1_ is not needed. Another potential solution could be to fit for the inversion efficiency, however this requires the use of an SPGR readout with reduced SNR efficiency a four parameter fit that is more sensitive to noise [14]. A shorter adiabatic pulse could also be used to improve the inversion efficiency, although this is often limited by the maximum allowable RF transmit power [15].

Other potential factors limiting MOLLI accuracy in vivo, but not studied here, were reviewed in [13] and may include, but are not limited to deviation from the nominal flip angle profile [24], [26], heart rate variability [18], [27], [28], magnetization transfer (MT) effect [26], [29]–[31], motion [19], and B_0_ inhomogeneity [13], [32]. Recent works have proposed acquiring MT sensitive data (e.g., by varying RF pulse length) and including MT effect in the signal model [26], [29], [30] for accurate T_1_ fitting; however, the utility of this approach for cardiac T_1_ mapping using MOLLI remains to be investigated. A potential solution for these imperfections could be to develop a IR-FSE based T_1_ mapping sequence [22] which is more robust against imperfect inversion efficiency and field inhomogeneities than the bSSFP based MOLLI sequence.

In conclusion, the conventional correction (Eq.1) of apparent T_1_ in MOLLI can lead to significant in vivo T_1_ errors partly due to lower adiabatic inversion efficiency in muscle tissues. T_1_ errors can be reduced significantly by using a modified version of the conventional MOLLI correction accounting for inversion efficiency (Eq.2), when the inversion efficiency is known.
